# Letter from C. C. Allen, Dentist, of New York

**Published:** 1846-03

**Authors:** Chas. C. Allen


### ARTICLE VI.

### *Letter from* C. C. Allen, Dentist, of New York.

Messrs. Editors:

As one of the objects of the Dental Journal is to discuss all subjects, intimately connected with the dental art, I beg leave to submit the following remarks upon an article in the last number, entitled, "Resolutions of the American Society of Dental Surgeons, in relation to the use of amalgams for filling teeth. First, let me inquire what was the ground upon which the *American Society of Dental Surgeons* first took their action against amalgams? It was not that a majority believed that amalgams were in all, or in a very large proportion of cases in which they are carefully used, productive of bad results, either to the teeth or constitution. They did not believe that great injury was done to teeth by members of their society, for those who used them, so far as I know in this city, were among the best and most skilful operators in the society. Nor did they intend to apply the epithet of quack to well educated and honest dentists who occasionally used amalgams. They had seen that there was a large class of men in the country, calling themselves dentists, who, either through ignorance or empiricism, were using and recommending amalgams as better than gold or tin, in cases were only gold was admissible, and that these men were doing great injury to their patients, and bringing reproach upon the dental science and upon all practicing dentists, the honest and intelligent, as well as the ignoramus and empiric. It was a measure of self-defence; they saw that as a whole, taking the operations of the good and the bad together, the amalgam was doing infinitely more hurt in the hands of the latter, than good in those of the former, and like the reformers in the noble cause of temperance, they resolved that henceforward they would have nothing to do with it. Substantially this has been the uniform answer of every member with whom I have spoken upon the subject, and who has taken any part in opposition to the use of amalgams for stopping teeth; and I appeal to each member of the society to know if it was not so.

Such then was the feeling of the society up to the time of its meeting in New York, last August. Many of the members at that time, seeing that the influence of the American Society of Dental Surgeons had not stopped the use of amalgams, but that it was rather on the increase, thought it necessary to take some more decisive *action* upon the subject. The course which the society pursued at that time, I will not now detail, as I presume it is known to all the readers of this Journal. It is sufficient to say, that most, if not all of the members present at that meeting, I believe there was not a single exception, pledged themselves to the society, to discontinue the use of amalgams in their practice as dentists.* Several who *believed* it to be useful, in some extreme cases, for stopping teeth, made this concession for the sake of promoting peace and harmony in our society. Here they supposed the matter would rest, and that amalgam, so far as the society was concerned, was virtually dead

* The committee appointed by the society last August, reported, "that six members in New York, used the amalgam, under certain circumstances and refused to discontinue its use." Now, although three out of the six, viz. John Lovejoy, George E. Howes and Chas. C. Allen, subsequently communicated to the society their intention to discontinue its use for the future, yet in the report of the proceedings of the society, published in the September number of the Journal, no mention is made of this fact. Their names still appear in the published proceedings, among the names of those who refuse to give up the use of amalgam. This is, manifestly, unfair.

and buried, when, just as we were congratulating ourselves upon the restoration of harmony among the different members, lo! its ghost appears in the form of a protest, extended to each member, and *demanding* his signature, not only pledging himself not to use amalgam, but also declaring that, in his *opinion*, it is in all cases "*unfit* and *dangerous*, when used for plugging the teeth or their fangs." Did any member suppose that ghost would frighten honest men and compel them to subscribe to what they believed to be a *lie*? Can a majority of three-fourths be found, to expel them for refusing to do it? Has the minority in this case no rights? Is there no alternative left to them but to resign or violate their own reason and conscience, whenever it happens that they cannot vote with the majority? I answer, there is. The minority have rights as well as the majority, and they are guaranteed to it by the original compact under which the society was formed.

Second. Let us see what the *constitution* says upon this subject. It will not, I presume, be denied by any member of our society, that the first law of our association, under which it was formed, and without which it could not well exist, is the *constitution*, and that all the provisions contained therein are inviolate and cannot be changed, except in the manner prescribed therein. If this position be true, then it follows that no resolution passed by a majority, I care not how large, is of any binding force upon the members, if the *principle* contained in the said resolution conflicts with the *principles* laid down in the constitution.*

In the preamble of the constitution is the following declaration, viz. that one of the objects of the American Society of Dental Surgeons, is, "to advance the science (of dentistry) by free communication and interchange of *sentiments*, either written or verbal, between members of the society, both in this and other countries." Does not this clause secure to each member the right to advocate any principle or "sentiment" connected with

* The constitution is the act of the people, speaking in their original character, and defining the pernament conditions of the social alliance, and there can be no doubt on this point with us, that every act of the legislative power, contrary to the true intent and meaning of the constitution, is absolutely null and void.—*Kent's Commentaries.*

dentistry, if done in an honest and proper manner? Again, in article 4th, upon the expulsion of members, the constitution reads thus: "Any member of the society may be expelled for immoral conduct, malpractice in business or other sufficient cause, on motion of one member, seconded by another, at any *regular meeting* of the society; in which case a majority of three-fourths of the members present, shall be required."

Now, those members of the society who refuse to give their testimony, that amalgams are in all cases "unfit and dangerous when used for plugging teeth or their fangs," were either expelled at the last "*regular meeting*," or they were not, if they were expelled, then the simple act of signing a protest cannot restore them; but if they were not expelled, then they cannot be until the next "*regular meeting*." There can be no *constitutional* expulsion, the object of the above article being, plainly, to bring the society to a direct vote upon the expulsion of each member.

Entertaining these views, I cannot help agreeing with your correspondent, who declares, that "the society has certainly transcended its powers;" but you remark: "The opinion that the use of all amalgams is malpractical, had stood recorded, not only upon the book of record, but in the Journal, the organ of the society, for four years, at the time the circular was ordered. This opinion every man was as much bound to adopt as any precept in either the constitution or by-laws." Now, suppose a resolution were to be passed by the society, that in its opinion, hereafter, the society had better meet every year at Baltimore instead of New York, and that one of you, gentlemen, being a northern man, should vote in the negative. Would you be as much bound to adopt this *opinion* as any precept in either the constitution or by-laws? Would you not rather say, "I have a right to remain in the society and enjoy my own opinion, but, nevertheless, for the sake of union and harmony, as a large majority of the society desire it, I will concede this point and go to Baltimore?"

Again you ask, "Is it not true that any position which would prevent one's obtaining membership, ought to deprive him of membership, in any voluntary association?" I answer, no.

The admission and expulsion of members, are two very different things. Hereafter, the members may, if they choose, ascertain the opinion of each candidate for admission to the society, upon the subject of amalgam and its fitness or unfitness for plugging teeth, and if they deem it heterodox they may vote against him; but as this has not, heretofore, been made a *test question*, it will not apply to actual members.

The candidate for admission to a christian church subscribes to the creed of articles of faith, as a matter of course, as members do to the constitution and by-laws of our society; but once a member, if a majority vote to exclude wine from the communion table, would he not be allowed to differ in opinion from the majority so voting, and, if in good standing in other respects, would the church excommunicate him because he could not believe with the majority upon this minor point, after he had conceded so much as to abandon the use of wine under all circumstances? You would make the opinion of a majority of a church, upon every subject which might come up, of the same binding force as any precept contained in the creed or articles of faith.

Third. In my opinion, the resolutions passed by the American Society of Dental Surgeons, and embodied in their protest against amalgams, were not only arbitrary, but unwise and exceeding injudicious. They establish a precedent fraught with innumerable evils, which will be productive of constant trouble to the society. For instance—grant that all are expelled who refuse to say that amalgam "is not only unfit, but dangerous, when used for plugging teeth or their fangs." Carry out the same principles, and another batch will probably be expelled, whenever the society see fit to correct the abuse of arsenic—an article which is, at this moment, probably doing as much mischief as amalgam—not because they do not know how to use the article; but because there are hundreds who abuse it. Again, the turnkey is a very imperfect instrument for extracting teeth compared with a good set of forceps; what shall prevent the society, at some future day, from passing a resolution that the turnkey is not only unfit, but dangerous, when used for extracting teeth or their fangs, and from expelling all the members who refuse to subscribe to this opinion?

Is this the way "to promote union and harmony among all respectable and well informed dental surgeons," or will such proceedings "give character and respectability to the profession?" Is it not rather the way to drive all honorable and intelligent men from the society? No man, who thinks for himself, can hope to remain long in any society, and always agree with the majority; and if this system is to be began and perpetrated, how long before the American Society of Dental Surgeons will be reduced to only a quorum of members, and a part of these be found voting against the rest?

This is the direct and legitimate result to which the society must come, if it continues to proscribe members for opinion's sake. What then is to be done? you ask, and I will endeavor to answer. In the first place, I would not have our society pronounce any operation, malpractice *a priori;* but make each member responsible, for his practice, to the society. If there is a member whose operations are injurious to his patients or bring disgrace upon the profession, let him be reported to the society, whether it be with amalgam, arsenic, the turnkey, or any other article, give him an opportunity to defend himself, and if the charges are proved against him, let him be expelled.

Such, I believe, is the course pursued in all medical and surgical societies, and there is less difficulty in proving malpractice in dentistry, than in medicine or surgery, as it will always show for itself, so long as the tooth stands. One of the principal medicines in the practice of the "steam doctors," is *lobelia inflata*, or Indian tobacco, a powerful and searching emetic, the operation of which, when injudiciously administered, often produces fatal results; but we never hear of any medical society pronouncing the use of this article to be malpractice—on the contrary, many eminent physicians believe it to be a valuable therapeutic agent when properly administered.

I would have our society put amalgams, arsenic, kreosote, and every other article used in dentistry, upon the same footing. Let time correct the abuse of all, as it has that of the file, which, not many years since, was often used by quacks for separating sound teeth to prevent them from decaying. I do not know of a member of our society who is not a good operator and an honorable man, worthy to be trusted with any of the above named articles. Surely, in this city, those members who have used the amalgam, will compare favorably with those who have not. Now, if there is one who meets with a tooth which he thinks he can fill better with amalgam than with gold, let him use it, and take the responsibility. Since the days of Paracelsus there have been an abundance of quacks, and the history of medicine shows, too plainly, that all proscriptive measures have rather increased than lessened their influence. The people soon find them out and avoid them; but when once in our zeal we go beyond the truth, in attempting to put them down, the cry of persecution will soon create a powerful reaction in their favor.

If the American Society of Dental Surgeons would suppress empiricism in one profession, let it devote all its time and energies to a diffusion of a knowledge of the principles of dentistry among all classes of society, and particularly among members of the profession; but let it not waste its strength in domestic feuds, nor its amunition in a petty warfare against an enemy who will flee like the mists of morning, whenever the light of truth and science shall illumine its dark and mischievous hiding places.

**CHAS. C. ALLEN, M. D.**

